# Bacteremia Identified After Emergency Department Discharge in Adults: A Retrospective Analysis of Outcomes and Risk Factors

**DOI:** 10.3390/antibiotics15090877

**Published:** 2026-09-08

**Authors:** Orit Wolfovitz Barchad, Ido Weinstock, Yonit Wiener-Well, Eli Ben-Chetrit

**Affiliations:** Infectious Disease Unit, The Eisenberg R&D Authority, Shaare Zedek Medical Center, Faculty of Medicine, The Hebrew University of Jerusalem, Jerusalem 9112102, Israel

**Keywords:** bacteremia, emergency department, hospital discharge, readmission, blood culture, risk stratification

## Abstract

Background: Adults who undergo blood-culture sampling in the emergency department (ED) may be discharged before results are finalized and later found bacteremic. Factors identifying which patients require readmission are not well defined. We sought predictors of return and hospitalization available at the index ED visit. Methods: In this retrospective study at a 1000-bed university-affiliated hospital (September 2021–March 2026), we included adults discharged from the ED with a positive blood culture identified post-discharge. The primary outcome was ED return with hospitalization within 30 days. Multivariable logistic regression was restricted to variables available at the index ED visit. Results: Of 197 eligible patients, 16 were excluded, leaving 181 analyzed: 78 (43.1%) were managed as outpatients, 36 (19.9%) returned and were re-discharged, and 67 (37.0%) returned and were hospitalized. Median age was 73 (IQR 61–83) years and 105 (58.0%) were male. Index-visit vital signs and laboratory values did not differ across trajectories. The only independent predictor of return and hospitalization was a non-UTI working discharge diagnosis (aOR 2.53, 95% CI 1.3–5.0). Creatinine ≥ 2 mg/dL (aOR 2.65, 95% CI 0.9–7.5) and previous hospitalization within 6 months (aOR 1.82, 95% CI 0.8–4.1) were significant per univariate analysis but attenuated after adjustment, and age was not predictive. Enterobacterales predominated (n = 111, 61.3%). All nine endovascular-infection cases were hospitalized. Overall mortality was 1.1% (2/181). Conclusions: Post-discharge bacteremia carried low mortality but frequent ED return and hospitalization. A non-UTI working diagnosis independently identified a higher-risk minority; renal impairment and recent hospitalization were supportive but non-independent markers.

## 1. Introduction

Febrile illness is commonly encountered in emergency departments (EDs), often requiring blood cultures to diagnose serious infections. Blood culture results usually indicate a significant infection but require 24–48 h for bacterial growth detection. Most labs incubate cultures for up to 5 days before discarding them if no growth is detected [1]. Therefore, patients might be discharged before culture results are available, even if initial evaluations warranted culture collection.

Recent trends show a decrease in hospital admissions despite more complex patient cases [2]. This may be attributed to better outpatient care, and the high cost and potential harm of unnecessary hospitalizations. In Israel, admissions for patients aged 15 or older dropped by 29% from 2010 to 2022 [3]. Subsequently, the risk of discharging bacteremic patients increases.

ED physicians often learn of positive blood cultures for patients discharged days earlier, raising concerns about clinical judgment, care quality, and legal implications. While discharge of children with bacteremia (most frequently, occult bacteremia with *Streptococcus pneumoniae*) is well acknowledged [4,5,6], there is less consensus on this issue with respect to adults. Previous reports have demonstrated that early discharge of adults with bacteremia has been associated with factors like normal vital signs and low CRP, and is generally reported to have a benign course [7,8,9,10].

This study aimed to determine the clinical profile and outcomes of adult patients (aged 18 years or older) discharged from the ED with bacteremia identified post-discharge. Additionally, we aimed to identify factors associated with ED readmission among these patients. We hypothesized that bacteremic patients discharged from the ED who did not return and require hospitalization would demonstrate a younger age, a lower level of CRP, and a greater prevalence of urinary tract infection as the source of bacteremia, when compared to those requiring hospitalization.

## 2. Results

### 2.1. Study Cohort and Disposition Trajectories

**Figure 1:**
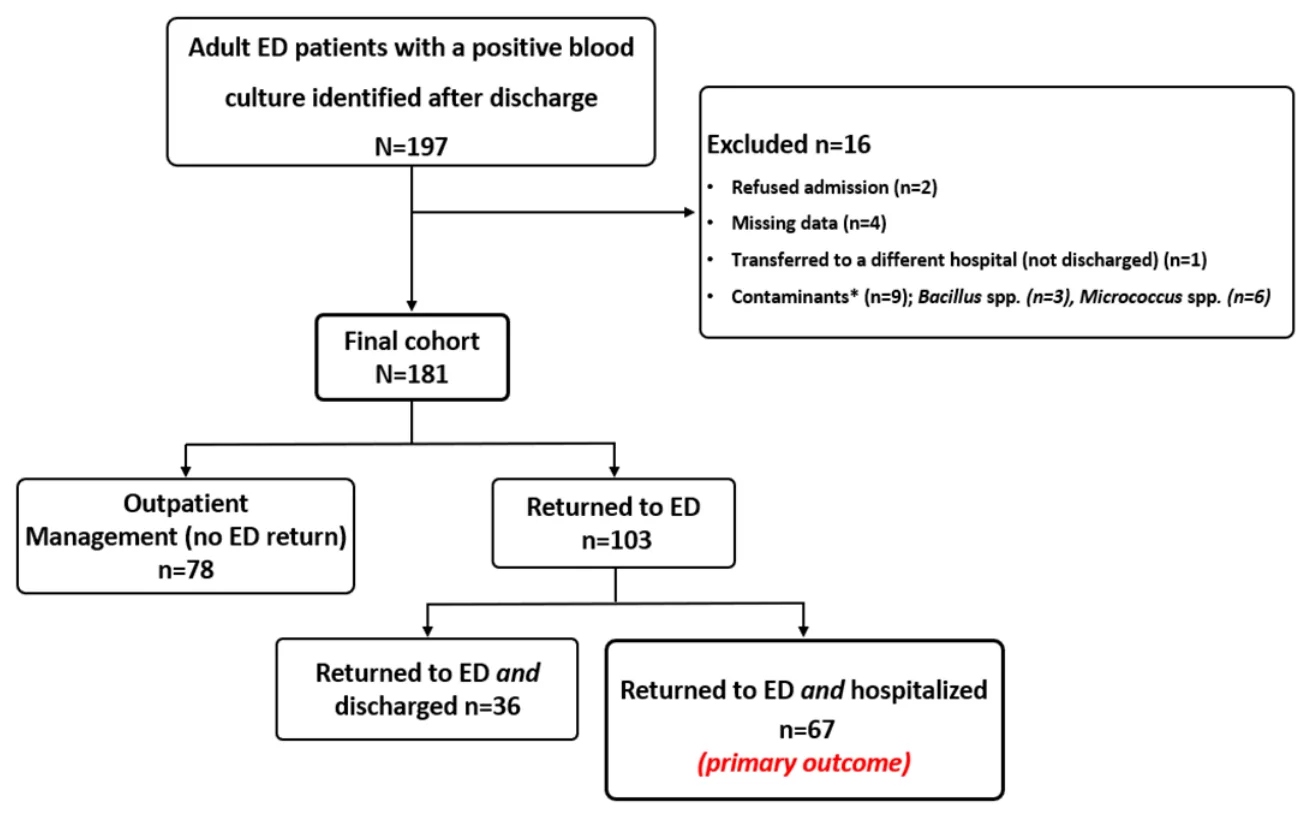
Derivation of the study cohort. * Contaminants excluded at this stage comprised *Bacillus* spp. (n = 3) and *Micrococcus* spp. (n = 6). Coagulase-negative staphylococci growing in a single bottle were classified as contaminants in accordance with institutional practice and were not recorded as clinically significant positive cultures; these cases therefore do not appear in this flow diagram. ED, emergency department.

During the study period, 373,385 patients presented to the ED and 47,737 blood culture sets were obtained; 4635 (9.7%) were positive, and 1504 sets (3.2% of all sets drawn) were judged to represent contamination, almost all coagulase-negative staphylococci. At the patient level, 197 adults were discharged from the ED before a clinically significant positive culture was identified; 16 were excluded, leaving 181 for analysis (Figure 1). Following discharge, 78 (43.1%) were managed as outpatients (no ED return), 36 (19.9%) returned and were re-discharged, and 67 (37.0%) returned and were hospitalized (the primary outcome). Overall, none of the patients returned on their own initiative before contact. Thirty-day all-cause mortality was low (two of 181 patients, 1.1%). Both were nonagenarians with skin/soft-tissue- or bone-source bacteremia—one discharged and one hospitalized.

### 2.2. Baseline Characteristics

Table 1 summarizes demographic and clinical characteristics for the overall cohort and by ED disposition trajectory. Median age was 73 years (IQR 61–83), 105 patients (58.0%) were male, and the median Charlson Comorbidity Index was 4.0 (IQR 3.0–6.0). Age, sex, overall Charlson Comorbidity Index, Norton score, and index-visit vital signs did not differ significantly across the three trajectories.

One baseline factor differed significantly across trajectories: patients who ultimately returned and were hospitalized were less likely to have a suspected urinary source of infection [28.4% (n = 19) vs. 55.1% (n = 43) and 38.9% (n = 14) among non-returners and returned-and-re-discharged patients, respectively; *p* = 0.005, Table 1]. Previous hospitalization within the past 6 months showed a similar trend [29.9% (n = 20) vs. 14.1% (n = 11) and 16.7% (n = 6)] with borderline significance (*p* = 0.053). Moderate/severe renal failure or dialysis was numerically more frequent in the hospitalized group but did not reach significance and involved few patients [7.5% (n = 5) vs. 1.3% (n = 1) and 2.8% (n = 1); *p* = 0.15].

### 2.3. Laboratory Findings at the Index Visit

Table 2 shows index-visit laboratory values by disposition trajectory. No continuous laboratory value—white cell count, neutrophil percentage, platelet count, CRP, or lactate—differed significantly across the three trajectories. The proportion of patients with creatinine ≥ 2 mg/dL was higher among those hospitalized (17.2% vs. 3.8% and 11.1%; *p* = 0.031). Among returners, the second-visit CRP was higher in those who were hospitalized than in those discharged again (median 12.9 [IQR 7.2–22.4] vs. 9.5 [IQR 5.8–16.7] mg/dL), but this difference did not reach statistical significance (*p* = 0.07). Index-visit CRP did not differ between these groups (median 5.1 [IQR 2.4–10.0] vs. 5.3 [IQR 1.4–12.3] mg/dL, *p* = 0.84).

### 2.4. Microbiology

**Figure 2:**
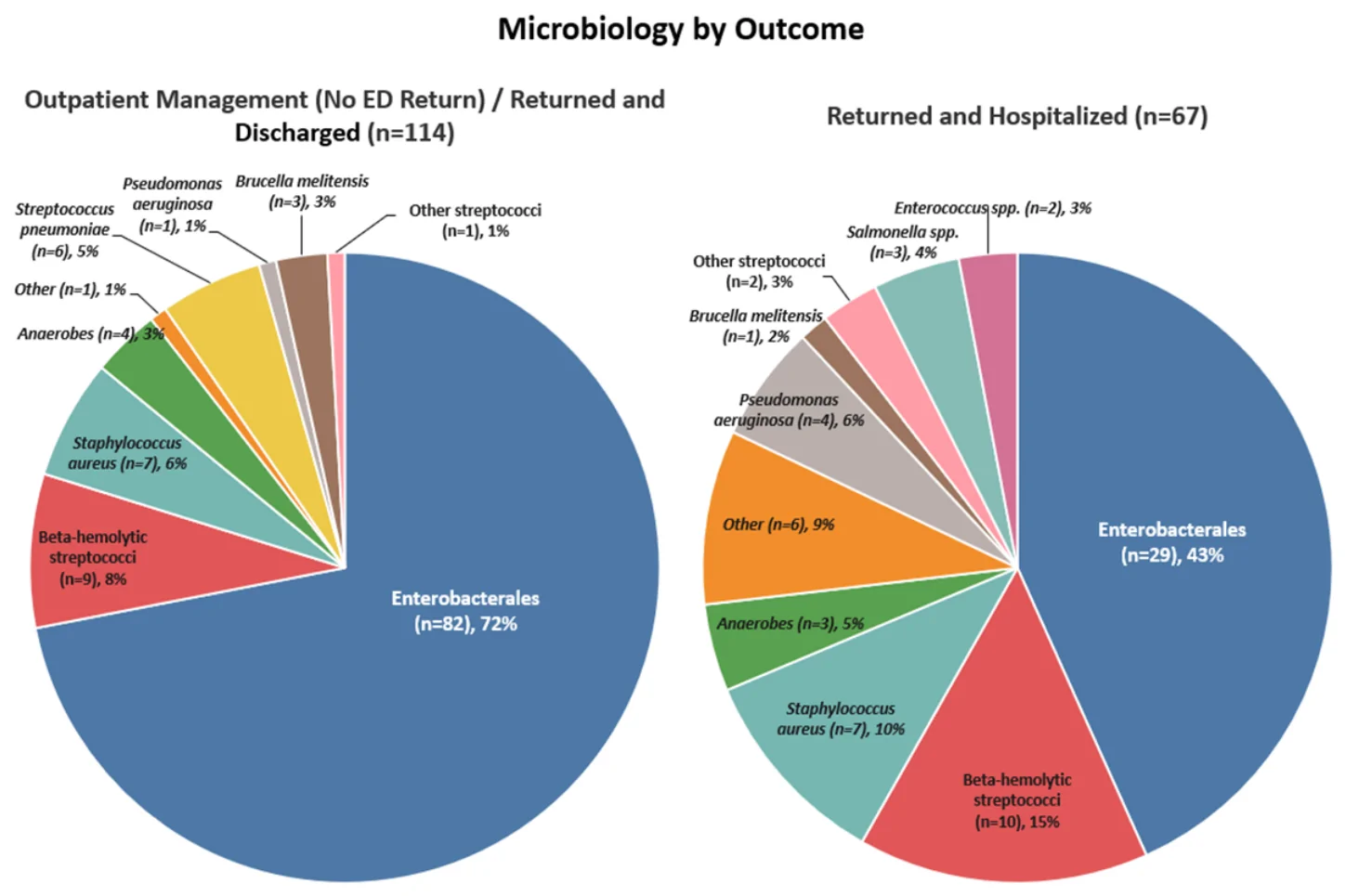
Distribution of pathogens by disposition outcome: no hospitalization vs. hospitalization. Enterobacterales were significantly more common in non-hospitalized patients (72% vs. 43%, *p* = 0.0002).

With regard to microbiology, Enterobacterales (predominantly *Escherichia coli*) accounted for 111 of 181 isolates (61.3%), followed by β-hemolytic streptococci (19 isolates, 10.5%) and *Staphylococcus aureus* (14 isolates, 7.7%). The distribution of pathogens differed between patients who were hospitalized on return (n = 67) and all others (n = 114, comprising non-returners and those returned then re-discharged); Enterobacterales predominated in the latter group but accounted for a smaller share among those hospitalized (72% vs. 43%, *p* < 0.001) (Figure 2).

### 2.5. Factors Associated with Return and Hospitalization

Table 3 compares baseline characteristics between patients who returned and were hospitalized (n = 67, the primary outcome) and all other patients (n = 114). Significant univariate associations included previous hospitalization in the past 6 months (OR 2.43, 95% CI 1.2–5, *p* = 0.02) and creatinine ≥ 2 mg/dL (OR 3.17, 95% CI 1.2–8.7, *p* = 0.02); moderate/severe renal failure or dialysis was directionally similar but did not reach significance (OR 4.52, 95% CI 0.9–24, *p* = 0.08). No index-visit vital sign reached significance individually. A UTI working discharge diagnosis was protective against return and hospitalization (OR 0.40, 95% CI 0.2–0.8, *p* = 0.005).

**Figure 3:**
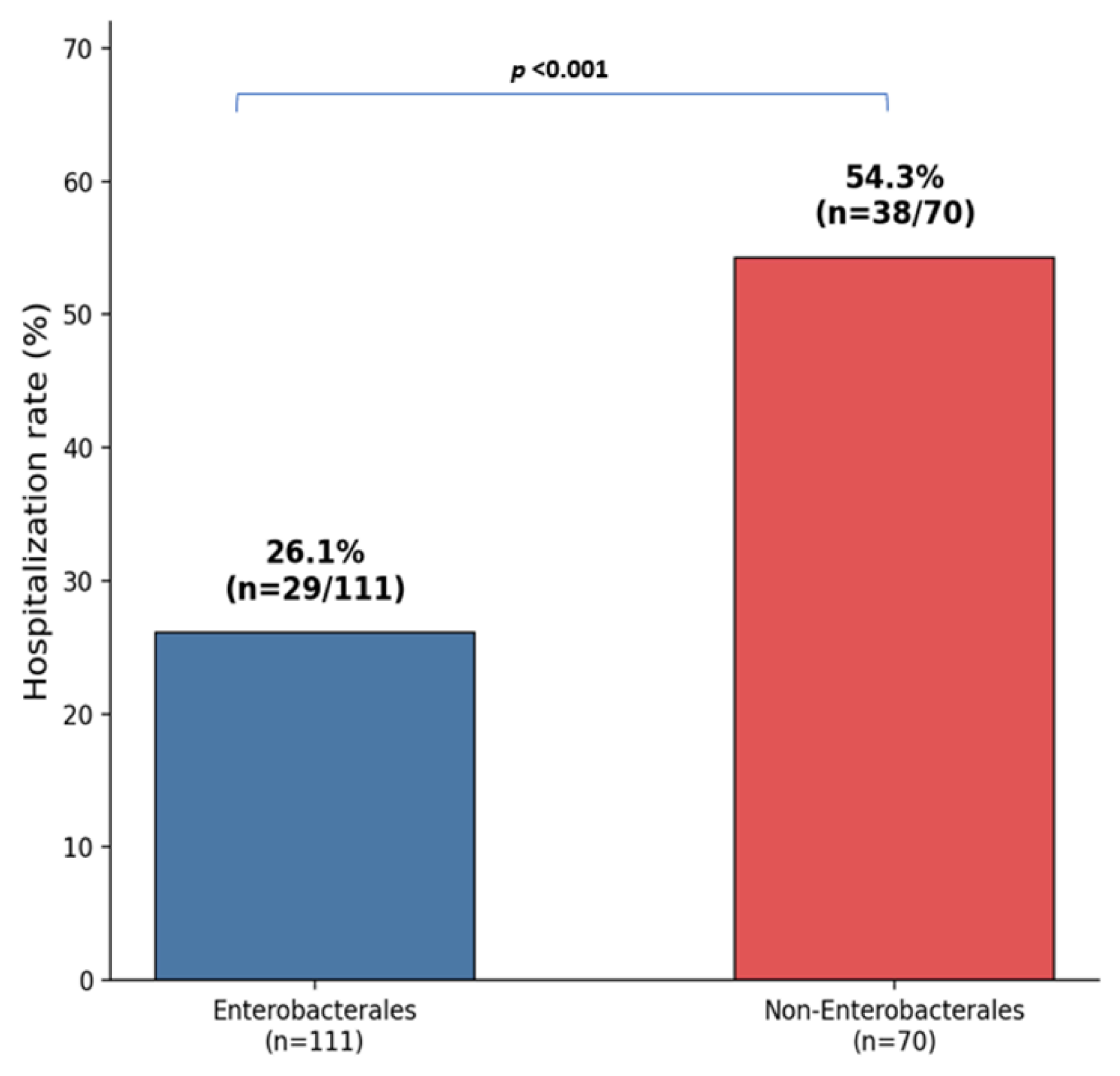
Hospitalization rate by organism group (Enterobacterales vs. non-Enterobacterales).

Four variables that became known only after the index visit were associated with the primary outcome: a post-discharge diagnosis of urosepsis, which was protective against return and hospitalization (OR 0.34, 95% CI 0.2–0.6, *p* < 0.001; Table 4), *Enterobacterales* bacteremia relative to all other organisms (OR 0.30, 95% CI 0.16–0.56, *p* < 0.001; Figure 3), and abdominal or endovascular infection (see below).

**Figure A1:**
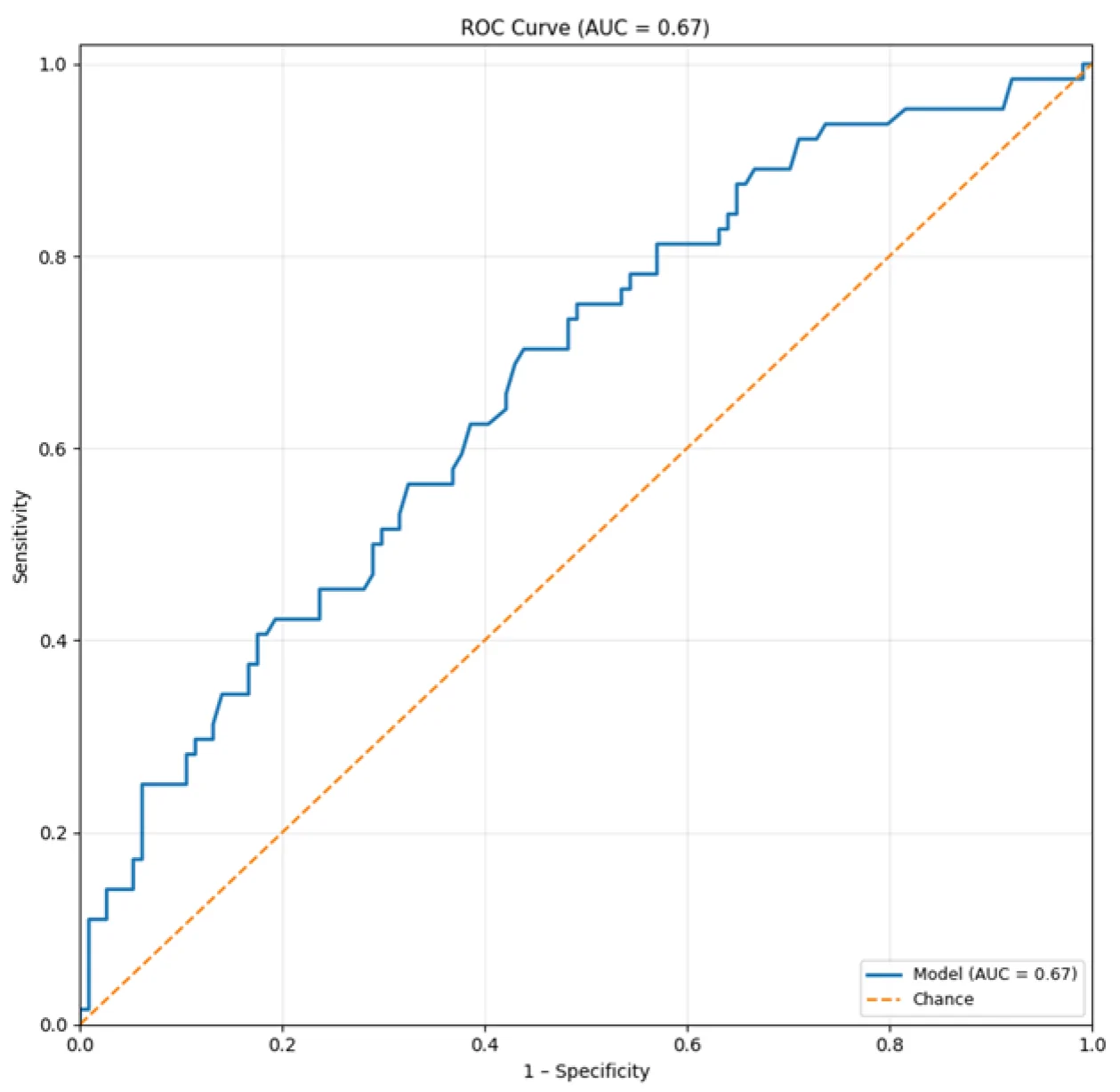
Receiver operating characteristic (ROC) curve for the multivariable model predicting return and hospitalization (N = 178, 64 events). Three patients with missing creatinine values, all in the hospitalized group, were excluded from the model. AUC 0.672 (95% CI 0.59–0.75); optimism-corrected AUC 0.643 by bootstrap validation.

A multivariable logistic regression model was constructed using only index ED visit variables including age, previous hospitalization, creatinine ≥ 2 mg/dL, and a non-UTI working discharge diagnosis (Table 5). The model was fitted on 178 patients with complete data for all four predictors (64 events); three patients, all in the return-and-hospitalization group, were excluded because of a missing creatinine value. Only a non-UTI working discharge diagnosis was independently associated with the primary outcome (aOR 2.53, 95% CI 1.29–4.96, *p* = 0.007). Creatinine ≥ 2 mg/dL (aOR 2.65, 95% CI 0.93–7.54, *p* = 0.068) and previous hospitalization (aOR 1.82, 95% CI 0.82–4.06, *p* = 0.14) did not retain independent significance; age was not predictive (aOR 0.97 per decade, *p* = 0.67). Model discrimination was modest (apparent AUC 0.672). Internal validity was assessed by bootstrapping (2000 resamples; Steyerberg et al. [11]). The optimism-corrected AUC (0.643) was only 0.029 below the apparent value, indicating minimal overfitting. The ROC curve is shown in Figure A1 (Appendix A).

### 2.6. Endovascular Infection Subgroup

Nine patients (5% of the cohort) had a definite or likely diagnosis of endovascular infection (five endocarditis, four line-associated sepsis). All nine were called back to the ED and hospitalized. Compared with the overall cohort, these patients were younger (median age 57 vs. 73 years) and carried a distinctly non-*Enterobacterales* organism profile: *Staphylococcus aureus* (n = 3), *Pseudomonas aeruginosa* (n = 2), a HACEK organism (*Aggregatibacter actinomycetemcomitans*, n = 2), a viridans-group streptococcus (*Streptococcus mitis/oralis*, n = 1), and *Staphylococcus epidermidis* (n = 1). Notably, eight of the nine (89%) had indwelling hardware (intravascular catheters, prosthetic valves, or cardiac devices).

### 2.7. Exploratory Analyses

The index-visit working diagnosis was concordant with the eventual culture-confirmed source in 107 of 181 patients (59.1%); the remaining 74 (40.9%) were discharged without a correct source diagnosis (Table A1, Appendix A). Hospitalization on return was more than twice as frequent among these missed cases than among concordant cases [41/74 (55.4%) vs. 26/107 (24.3%)]; risk ratio 2.28, 95% CI 1.54–3.37; risk difference 31 percentage points. This excess was not distributed evenly: abdominal, skin/soft-tissue-and-bone, and endovascular sources together accounted for 31 of the 41 hospitalizations (76%) in the missed group, whereas missed urinary-source cases carried a substantially lower admission rate (8/24, 33%). 

**Figure A2:**
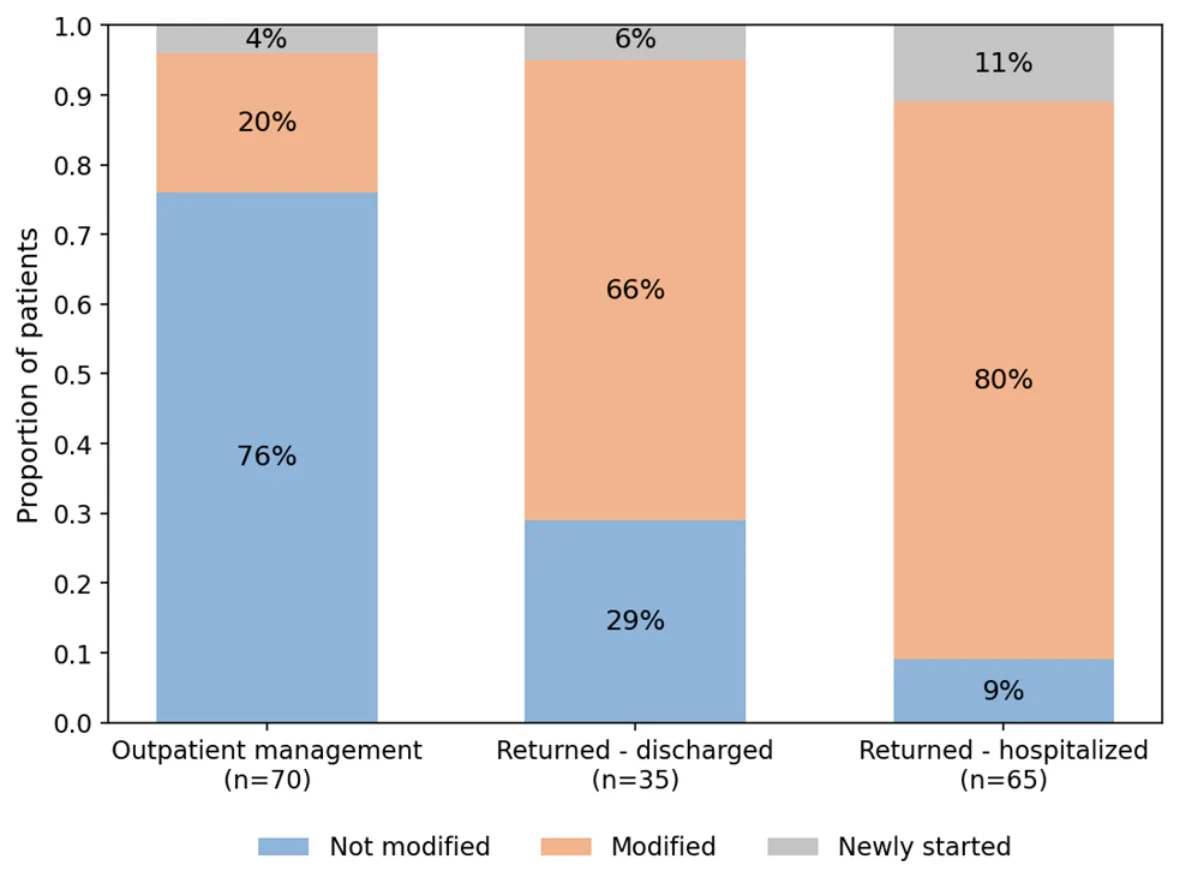
Antibiotic modification or initiation by trajectory. The proportion of patients whose empiric therapy was already appropriate fell sharply across trajectories—from 75.7% to 28.6% to 9.2%—while the need for any change (modification or new initiation) rose correspondingly. Analysis restricted to 170/181 patients (94%) with documented antibiotic-modification data.

Overall, 158 of these patients (92.9%) received an antibiotic at the index ED visit, a proportion that was similarly high across all three disposition trajectories (95.7%, 94.3%, and 89.2%, respectively; Figure A2). Antimicrobial therapy was modified or newly started after culture notification in 101 of 170 patients (59.4%) (Figure A2, Appendix A). Regimen change was markedly more frequent among patients who returned and were hospitalized (59/65, 90.8%) than among those who returned but were discharged (25/35, 71.4%) or who did not return (17/70, 24.3%; Fisher’s exact *p* < 0.001), consistent with initial empirical coverage having been inadequate for the confirmed pathogen in the hospitalized group. These analyses were restricted to the 170 of 181 patients (94%) with documented data on antibiotic modification.

## 3. Discussion

In this retrospective cohort of 181 adults discharged from the ED with subsequently identified bacteremia, more than half (56.9%) returned and over a third (37.0%) were ultimately hospitalized. Of the factors assessed at the index visit, only a non-UTI working discharge diagnosis independently predicted this outcome; previous hospitalization and creatinine ≥ 2 mg/dL were significant on univariate analysis but did not persist after adjustment, while vital signs did not differ.

These findings are consistent with prior studies describing bacteremia in adults discharged from the ED as generally benign [7,8], with most patients following a favorable course. Miwa et al. [9] and Gonzalez-Del Vecchio et al. [8] similarly found that most bacteremic patients discharged from the ED did not require subsequent intervention.

Enterobacterales predominated, consistent with Kenig et al. [10], who reported the same group as the most common pathogens after ED discharge (67.0%); this predominance was more pronounced among patients who neither returned nor required hospitalization than among those hospitalized (72% vs. 43%). 

Index-visit vital signs did not predict return and hospitalization, likely reflecting the population itself: patients well enough to be discharged were, by definition, those without overt physiological derangement. Consistent with this, milder illness severity at presentation was linked to the discharge decision in both, the Japanese series (absence of hypotension) and the study by Chang et al. (lower Pitt Bacteremia Score) [9,12]. The absence of abnormal vital signs likely indicates lower disease severity and explains why these measures did not discriminate between groups.

Our hypothesis that a urinary source would predict a more favorable course was confirmed: a non-UTI working diagnosis carried roughly 2.5-fold-higher odds of return and hospitalization on univariate analysis and was the only independent predictor after adjustment (aOR 2.53, 95% CI 1.3–5.0) (Table 5). This is directionally consistent with the 56–vs-36% non-UTI rate reported by Kenig et al. [10] between readmitted and non-readmitted patients; our rates were comparable, if somewhat higher [48/67 (71.6%) vs. 57/114 (50.0%), Table 3]. This finding should not be conflated with diagnostic uncertainty alone: point estimates were similar for patients with a specific alternative diagnosis (e.g., soft-tissue infection, abdominal infection) and those with a non-specific presentation (fever without a source; Table 3), suggesting that a non-urinary source, rather than uncertainty *per se*, primarily drives this association—though our sample size limits precision, and a larger prospective study is needed to confirm this.

The ED working diagnosis matched the eventual culture-confirmed source in 59.1% of patients, and the 40.9% in whom it did not were more than twice as likely to return and be hospitalized (Table A1). This parallels the 44.2% diagnosis-revision rate reported by Kenig et al., with the excess risk concentrated in deep-seated sources—abdominal, skin/soft-tissue-and-bone, and endovascular infections—rather than urinary ones.

By contrast, the other two components of our a priori hypothesis were not confirmed. Age did not differ between trajectories (median 70–76 years, Table 1) and was not an independent predictor (aOR 0.97 per decade, *p* = 0.67). CRP levels were comparable between the three trajectory groups, with medians of 5.9, 5.3, and 5.1 mg/dL, respectively (Table 2). This differs from earlier reports associating low CRP and younger age with a benign course [7,8,9,10], and may reflect our older cohort (median age 73 years), a different outcome (a composite of return and hospitalization rather than readmission alone), or limited power to detect modest continuous-variable effects. An early index-visit CRP may also be falsely low and rise only over the subsequent 24–48 h; indeed, CRP became discriminating only at re-presentation, once the illness had evolved (*p* = 0.07), although high CRP by itself can trigger hospitalization. Overall, standard severity and inflammatory markers drawn at the index visit did not distinguish patients who would later require readmission.

Previous hospitalization within 6 months was associated with the outcome on univariate analysis (OR 2.43, 95% CI 1.16–5.06) but did not retain significance after adjustment (aOR 1.82, 95% CI 0.82–4.06). Although attenuated, this association is consistent with recent hospitalization being a well-recognized marker of comorbidity burden and healthcare exposure, both of which predispose to readmission and to a more complicated infectious course [13].

Renal impairment (creatinine ≥ 2 mg/dL) was associated with the outcome on univariate analysis (OR 3.17, 95% CI 1.16–8.65) but did not retain significance after adjustment (aOR 2.65, 95% CI 0.93–7.54); the wide interval reflects the small number of affected patients (18, 10.1%). The discordance between the non-significant continuous creatinine comparison and the significant ≥ 2 mg/dL threshold likely reflects a distinct high-risk subgroup with moderate-to-severe renal impairment, rather than a linear relationship between renal function and outcome. Reduced renal function has been linked to a higher risk of bloodstream infection: James et al. reported increasing risk with declining eGFR [14], and a population-based Norwegian study found reduced eGFR associated with both bloodstream infection and sepsis [15]. Impaired neutrophil function and cell-mediated immunity as well as humoral dysfunction—all associated with uremia may contribute [16]. Renal impairment may therefore warrant a lower threshold for cautious disposition even when other indicators appear reassuring.

The nine endovascular infections formed a small but distinct group: younger patients with a non-specific index presentation, all ultimately hospitalized, and eight of nine with indwelling hardware (intravascular catheters, prosthetic valves, or cardiac devices)—the near-opposite of the low-risk urinary/Enterobacterales phenotype—a mismatch that likely contributed to their initial under-recognition, since neither age nor presenting symptoms flagged them as high risk. This aligns with evidence on organism-specific risk stratification for endocarditis: Freling et al. showed that simple blood-culture parameters—organism identity, number of positive bottles, and time to clearance—can adjust the pretest probability of infective endocarditis before echocardiography [17]. Combining organism identity with indwelling hardware in the notification/callback pathway, rather than relying on non-specific presentation, could plausibly shorten time to diagnosis in this subgroup. This needs confirmation in larger studies, as the subgroup was too small for formal testing and the dataset lacks other key risk factors (e.g., intravenous drug use, known valvular disease).

Skin, soft-tissue, and bone infections were similarly under-recognized at the index visit (Table A1); a more deliberate skin, joint, and musculoskeletal examination in patients without an obvious source, particularly older adults, where localizing signs may be subtle, could improve diagnostic concordance for this source.

Thirty-day mortality was low (two deaths, 1.1%), consistent with prior reports (2.1% at 28 days [9]; 5.6% at 90 days [10]). This offers some reassurance that, with appropriate culture follow-up, most adults discharged with unrecognized bacteremia can be recalled and treated before serious harm occurs. However, low mortality does not equate to a benign course: over half of patients returned to the ED and more than a third were hospitalized, reflecting substantial healthcare utilization and, in some cases, meaningful morbidity despite survival.

Our findings are also relevant to the debate about blood-culture use in the ED. Because cultures in low-risk patients often have low yield and many false positives [18], there is growing interest in decision rules or machine-learning models to draw fewer of them [19]. Discharged patients are usually assumed to be low risk, but our data question that assumption: even among adults well enough to go home, cultures found real bacteremia, and more than a third were later recalled and admitted. Restricting cultures to patients who will be admitted would miss these cases, leaving the alternative of waiting for deterioration or a second febrile presentation—a worse option than culture-based recall. Consistent with this, the need to change empiric therapy rose sharply across trajectories, from 24.3% of patients who were managed as outpatients to 90.8% of those hospitalized (Figure A2); still, 42 of 101 patients requiring a change (41.6%) were managed without admission, indicating that a positive culture signals the need to reassess therapy, not necessarily to hospitalize, and supporting structured follow-up protocols [20].

### Limitations

This study has several limitations. The single-center, retrospective design limits generalizability and raises the possibility that patients re-presenting elsewhere were misclassified as non-returners, although this is unlikely, since most patients return to the discharging hospital; consistent with active follow-up, the only out-of-Jerusalem admission we recorded followed our own contact with the patient (group A streptococcal bacteremia). Importantly, our primary outcome—return to the ED followed by hospitalization—is also not a purely objective measure of clinical deterioration. It reflects the sequential decisions of the ID consultant, the treating ED physician, and prevailing admission practices, any of which could vary independent of the patient’s underlying clinical trajectory. Some hospitalizations may reflect a cautious threshold to admit (e.g., in an older patient with newly recognized bacteremia) rather than objective deterioration, and conversely some patients managed as outpatients may have had unrecognized risk.

Sample size was modest (181 patients, 67 events), and predictors affecting few patients (e.g., renal failure or dialysis) yielded imprecise estimates with wide confidence intervals.

In addition, single-bottle CoNS isolates were treated as contaminants and not captured; true CoNS bacteremia, including device-associated infection, characteristically grows in both bottles and often in multiple sets [21], so few, if any, genuine cases are likely to have been missed.

Symptom duration prior to the index visit was not available either. Since CRP may lag clinical infection by 24–48 h, this could confound or modify the null laboratory findings.

Finally, lactate was missing in 46 of 181 patients (25%); because it is often measured selectively in sicker patients, these data may not be missing at random, potentially introducing bias.

## 4. Materials and Methods

### 4.1. Study Design and Setting

This observational retrospective study was conducted at the Shaare Zedek Medical Center (SZMC), a 1000-bed university-affiliated teaching hospital in Jerusalem. It included adult patients discharged from the ED between September 2021 and March 2026 who subsequently had positive blood cultures. The study was conducted in accordance with the Declaration of Helsinki and was approved by the Institutional Review Board (Helsinki Committee) of the Shaare Zedek Medical Center (approval no. SZMC-0011-25).

### 4.2. Blood Cultures and Microbiological Methods

At SZMC, blood cultures are drawn for patients with suspected bacterial infection, typically in the setting of fever. Each set includes both aerobic and anaerobic bottles. The BACTEC 9240 system (Becton Dickinson Diagnostic Instrument Systems, Sparks, MD, USA) processes these cultures for 5 days. Identification is done using Matrix-Assisted Laser Desorption/Ionization Time-of-Flight (MALDI-TOF, bioMérieux, Marcy-l’Étoile, France), and antimicrobial susceptibility testing is performed using the Kirby–Bauer disk diffusion method or Etest, and, since January 2026, the VITEK 2 automated system (bioMérieux, Marcy-l’Étoile, France).

### 4.3. Notification and Callback Pathway

Every new positive blood culture is logged in the microbiology laboratory (patient name, identification number, date, and Gram stain result). Each morning, the infectious disease (ID) consultant reviews these results; cultures from patients already discharged are flagged, and the consultant reviews the patient’s records, contacts the treating ED physician, and provides treatment recommendations. Coagulase-negative staphylococci growing in a single bottle of two are routinely judged to represent contamination and are not pursued (see below). Nearly all patients discharged with bacteremia are contacted by an SZMC ED physician within 24 h of ID specialist review and recommendation. Depending on the organism and the patient’s condition, the treating team may manage the patient remotely (e.g., continuing or adjusting oral antibiotics by telephone) or recall them to the ED for reassessment. The decision to recall, and the subsequent decision to admit (hospitalize) or discharge, rests with the ED physician.

### 4.4. Inclusion and Exclusion Criteria

The cohort consisted of adult patients who had blood cultures sampled in the ED before discharge. Patients who were admitted or who died on arrival were excluded. Those with likely contaminants [coagulase-negative staphylococci (CoNS), *Bacillus* spp., *Corynebacterium* spp. (diphtheroids), *Micrococcus* spp., and alpha-hemolytic streptococci growing in one bottle of a two-bottle set or in one bottle of multiple two-bottle sets], were excluded. Isolation of these organisms in two or more separate cultures was adjudicated individually by an infectious disease specialist (O.W.B. or E.B.-C.), incorporating clinical context (e.g., an indwelling device, immunosuppression, a compatible clinical syndrome); isolates judged clinically significant on adjudication were retained in the cohort, while those judged to represent contamination were excluded.

Patients discharged against medical advice, or with missing outcome data were excluded as well. All isolates—including those ultimately classified as contaminants—were reviewed by an infectious disease specialist (O.W.B., E.B.-C.).

### 4.5. Data Collection and Variables

We retrospectively reviewed the laboratory log records for the study period (September 2021–March 2026) to identify all adults who had been discharged from the ED before their blood culture became positive. Data collected included demographics (age, sex), Charlson Comorbidity Index, Norton score, vital signs (fever, pulse rate, lowest recorded systolic blood pressure, oxygen saturation), discharge diagnosis, empirical and definitive antibiotic regimens (including whether modified post-discharge or after readmission), and whether antibiotics were given at the index visit at all. Laboratory data included white blood cell count, platelet count, renal function tests, CRP, lactate, and blood culture details including organism identification and antimicrobial susceptibility testing.

A thorough review of the patients’ medical files was performed (for initial ED referral and after discharge) to assess outcome measures (see below).

### 4.6. Outcome Measures

Outcome measures were assessed over the 30 days following discharge, and included: return to the ED, with disposition classified as either hospitalization or discharge; modification of the antimicrobial regimen following culture notification; and 30-day mortality. The primary outcome for the analyses in this report was a composite of return to the ED and subsequent hospitalization, distinguishing patients who returned but were re-discharged from those whose return resulted in hospitalization.

In a post hoc analysis, we compared the distribution of causative organisms by disposition (hospitalized versus non-hospitalized on return). Two pre-specified secondary endpoints were also examined. First, we assessed the accuracy of the index-visit working diagnosis by classifying each patient as concordant or discordant with the post-discharge, culture-confirmed likely diagnosis (‘missed’ cases) and relating this to the primary outcome. Second, we described modification of antimicrobial therapy after culture notification (regimen unchanged, modified, or newly started) and its distribution across disposition trajectories.

### 4.7. Statistical Analysis

Continuous variables were presented as median (interquartile range, IQR), reflecting their non-normal distributions, and were compared using the Mann–Whitney U test (two groups) or Kruskal–Wallis test (three groups). Categorical variables were presented as n (%) and were compared using the chi-square test or Fisher’s exact test, as appropriate. Selected continuous vital-sign and laboratory variables were additionally dichotomized using standard clinical thresholds to aid interpretation.

Candidate predictors of the primary outcome were first screened using univariate logistic regression. A multivariable logistic regression model was then constructed, restricted to variables available at (or derived from) the index ED visit, so that the resulting model could inform risk stratification at the point of discharge. The four candidate variables, all available at the index visit, were entered simultaneously (enter method). These were selected using a combination of clinical and statistical criteria. Age was included *a priori* for face validity, given its established relevance to infection risk and outcomes in the general literature, irrespective of its univariate significance in this cohort. Previous hospitalization, creatinine ≥ 2 mg/dL, and a non-UTI working discharge diagnosis were selected because each was significantly associated with the primary outcome on univariate screening and each is ascertainable at the point of ED discharge.

Model discrimination was quantified as the area under the receiver operating characteristic curve (AUC); internal validation used bootstrap resampling (2000 resamples) to obtain an optimism-corrected AUC, following the approach of Steyerberg et al. [11].

Agreement between the working (pre-culture) discharge diagnosis and the post-discharge (post-culture) diagnosis was assessed descriptively as the proportion of patients in whom the two classifications matched (observed percent agreement). Each discharge diagnosis was categorized a priori as source-concordant or discordant with the final microbiological diagnosis; discharge diagnoses that did not specify an infective source (e.g., ‘viral,’ ‘fever without source’) were classified as discordant. The association between diagnostic concordance and the primary outcome was examined using the chi-square test, with the effect expressed as an odds ratio and 95% confidence interval. All analyses were performed in IBM SPSS version 30.0; a two-sided *p* < 0.05 was considered statistically significant.

## 5. Conclusions

Taken together, these findings refine, rather than overturn, the view that most adults in this cohort do well after ED discharge with later-identified bacteremia: a non-urinary working diagnosis—with renal impairment and recent hospitalization as supportive markers—helps identify the minority at higher risk. However, no combination of factors reliably identified a subgroup safe to exclude from follow-up; they may better guide the intensity of reassessment than whom to contact. Whether selective follow-up could safely reduce this burden, and its effect on outcomes and cost, warrants prospective study.

## Figures and Tables

**Table 1:** Baseline characteristics of the study cohort, overall and by disposition trajectory.

Characteristic	Overall (N = 181)	Outpatient Management, n = 78	Returned and Discharged, n = 36	Returned and Hospitalized, n = 67	*p* Value
Age, years (median, IQR)	73 (61–83)	76 (65–85)	70 (46–82)	71 (59–83)	0.2
Male sex, n (%)	105 (58.0%)	46 (59.0%)	18 (50.0%)	41 (61.2%)	0.6
Residence					0.8
Home	166 (91.7%)	72 (92.3%)	34 (94.4%)	60 (89.6%)	
Protected living	11 (6.1%)	4 (5.1%)	2 (5.6%)	5 (7.5%)	
Hospital	4 (2.2%)	2 (2.6%)	0 (0.0%)	2 (3.0%)	
Previous hospitalization, past 6 months	37 (20.4%)	11 (14.1%)	6 (16.7%)	20 (29.9%)	0.053
Charlson Comorbidity Index	4 (3–6)	4.5 (3–6)	4 (1–6)	5 (2–7)	0.3
Moderate/severe renal failure and/or dialysis	7 (3.9%)	1 (1.3%)	1 (2.8%)	5 (7.5%)	0.15
Diabetes mellitus	64 (35.4%)	30 (38.5%)	12 (33.3%)	22 (32.8%)	0.7
Congestive heart failure	33 (18.2%)	14 (17.9%)	3 (8.3%)	16 (23.9%)	0.15
COPD	7 (3.9%)	2 (2.6%)	1 (2.8%)	4 (6.0%)	0.5
Dementia	25 (13.8%)	15 (19.2%)	4 (11.1%)	6 (9.0%)	0.18
Solid tumor	27 (15.0%)	13 (16.9%)	4 (11.1%)	10 (14.9%)	0.7
Immunosuppression (chemo/steroids)	14 (7.7%)	5 (6.4%)	5 (13.9%)	4 (6.0%)	0.3
Norton score ^a^	19 (15–20)	19 (14.8–20)	19 (16–20)	18 (14–20)	0.5
**Index ED visit—vital signs**					
Fever (≥38.0 °C) ^b^	56 (32.7%)	23 (32.9%)	11 (31.4%)	22 (33.3%)	1
Heart rate, highest (bpm)	96 (82–108)	99 (83–110)	94 (85–105)	96 (80–108)	0.3
Systolic BP, lowest (mmHg)	120 (109–145)	124 (108–147)	119 (111–136)	120 (109–144)	0.8
Hypotension (SBP < 100 mmHg)	19 (10.5%)	7 (9.0%)	2 (5.6%)	10 (14.9%)	0.3
Hypoxia (SpO_2_ < 94%) ^c^	62 (35.0%)	30 (39.5%)	9 (25.0%)	23 (35.4%)	0.3
**Discharge diagnosis**					0.005
Urinary tract infection	76 (42.0%)	43 (55.1%)	14 (38.9%)	19 (28.4%)	
Pneumonia	25 (13.8%)	13 (16.7%)	5 (13.9%)	7 (10.4%)	
Soft-tissue, bone or joint infections	25 (13.8%)	8 (10.3%)	5 (13.9%)	12 (17.9%)	
Abdominal	10 (5.5%)	4 (5.1%)	2 (5.6%)	4 (6.0%)	
Viral	11 (6.1%)	0 (0.0%)	5 (13.9%)	6 (9.0%)	
Fever without a source	29 (16.0%)	10 (12.8%)	5 (13.9%)	14 (20.9%)	
Other ^d^	5 (2.8%)	0 (0.0%)	0 (0.0%)	5 (7.5%)	

^a^ n = 168; ^b^ n = 171; ^c^ n = 177; ^d^ Other diagnoses (n = 5) included otitis externa, atrial fibrillation, transient ischemic attack, anemia, and dehydration (one patient each). Continuous variables: median (IQR), Kruskal–Wallis test. Categorical variables: n (%), chi-square/Fisher’s exact test. BP, blood pressure; COPD, chronic obstructive pulmonary disease; ED, emergency department; IQR, interquartile range; SBP, systolic blood pressure.

**Table 2:** Laboratory values at the index ED visit, overall and by disposition trajectory.

Characteristic	Overall (N = 181)	Outpatient Management n = 78	Returned and Discharged, n = 36	Returned and Hospitalized, n = 67	*p* Value
WBC (×10^9^/L)	11.6 (8.6–14.6)	11.9 (8.7–15.2)	11.9 (8.6–15.2)	11.1 (8.5–13.6)	0.3
Leukocyte count (≤4 or ≥12 ×10^9^/L)	84 (47.2%)	40 (51.3%)	18 (50.0%)	26 (40.6%)	0.4
Neutrophils (%)	88.0 (79.7–91.8)	88.2 (79.8–91.5)	86.7 (78.7–92.8)	86.9 (79.8–91.4)	0.9
Platelet count (×10^9^/L)	204 (160–291)	214 (154–323)	208 (166–240)	198 (163–272)	0.5
Thrombocytopenia (≤150 ×10^9^/L)	40 (22.5%)	17 (21.8%)	8 (22.2%)	15 (23.4%)	1
CRP (mg/dL)	5.3 (2.1–11.4)	5.9 (2.4–14.1)	5.3 (1.4–12.3)	5.1 (2.4–10.0)	0.8
Lactate (mmol/L)	1.7 (1.2–2.3)	1.7 (1.3–2.4)	1.5 (1.0–2.0)	1.9 (1.1–2.4)	0.3
Creatinine (mg/dL)	1.0 (0.8–1.3)	1.0 (0.8–1.3)	1.0 (0.8–1.2)	1.0 (0.8–1.3)	0.9
Creatinine ≥ 2 mg/dL	18 (10.1%)	3 (3.8%)	4 (11.1%)	11 (17.2%)	0.031

WBC and platelets were missing for the same three patients (all in returned and hospitalized: 64/67 with data). CRP was missing for those three plus two more (77/78, 36/36, 63/67 with data). Lactate was missing in 46 patients (65/78, 25/36, 45/67 with data; 46/181 overall). All percentages use the non-missing N for that row/column. Continuous variables: median (IQR), Kruskal–Wallis test. Categorical variables: n (%), chi-square/Fisher’s exact test. CRP, C-reactive protein; WBC, white blood cell count.

**Table 3:** Univariate analysis of factors associated with return to the ED and hospitalization (primary outcome).

Variable	Outpatient Management/Returned and Discharged, n = 114	Returned and Hospitalized, n = 67	OR	95% CI	*p* Value
Age, years (per 10 years)	75.0 (63.0–83.8)	71.0 (58.5–83.0)	0.97	0.8–1.1	0.7
Age ≥ 65 years	81 (71.1%)	45 (67.2%)	0.83	0.4–1.6	0.6
Male sex	64 (56.1%)	41 (61.2%)	1.23	0.7–2.3	0.5
Previous hospitalization, past 6 months	17 (14.9%)	20 (29.9%)	2.43	1.2–5.0	0.02
**Comorbidities**					
Charlson Comorbidity Index (per 1 point)	4.0 (3.0–6.0)	5.0 (2.0–7.0)	1.03	0.9–1.1	0.6
Moderate/severe renal failure or dialysis	2 (1.8%)	5 (7.5%)	4.52	0.9–24	0.08
Diabetes mellitus	42 (36.8%)	22 (32.8%)	0.84	0.4–1.6	0.6
Congestive heart failure	17 (14.9%)	16 (23.9%)	1.79	0.8–3.8	0.1
Immunosuppression (chemo/steroids)	10 (8.8%)	4 (6.0%)	0.66	0.2–2.2	0.5
**Index ED visit—vital signs**					
Fever (≥38.0 °C)	34 (32.4%)	22 (33.3%)	1.04	0.5–2.0	0.9
Tachycardia (HR > 100)	45 (39.5%)	23 (34.3%)	0.80	0.4–1.5	0.5
Hypotension (SBP < 100 mmHg)	9 (7.9%)	10 (14.9%)	2.05	0.8–5.3	0.1
Hypoxia (SpO_2_ < 94%)	39 (34.8%)	23 (35.4%)	1.03	0.5–1.9	0.9
**Selected laboratory values (index ED visit)**					
Leukocyte count abnormal (≤4 or ≥12 ×10^9^/L)	58 (50.9%)	26 (40.6%)	0.66	0.4–1.2	0.2
Thrombocytopenia (Platelet count < 150 ×10^9^/L)	25 (21.9%)	15 (23.4%)	1.09	0.5–2.3	0.8
CRP ≥ 15 mg/dL	24 (21.2%)	9 (14.3%)	0.62	0.3–1.4	0.3
Lactate ≥ 2 mmol/L	33 (36.7%)	21 (46.7%)	1.51	0.7–3.1	0.3
Creatinine ≥ 2 mg/dL	7 (6.1%)	11 (17.2%)	3.17	1.2–8.7	0.02
Discharge diagnosis *					
UTI	57 (50.0%)	19 (28.4%)	0.40	0.2–0.8	0.005
Fever without a source	15 (13.2%)	14 (20.9%)	1.74	0.8–3.9	0.2
Pneumonia	18 (15.8%)	7 (10.4%)	0.52	0.2–1.4	0.3
Viral infection	5 (4.4%)	6 (9.0%)	2.54	0.8–8.4	0.1
Abdominal	6 (5.3%)	4 (6.0%)	1.14	0.3–4.2	1.0
Soft-tissue, skin or bone infection	13 (11.4%)	12 (17.9%)	1.70	0.7–4.0	0.3

* A seventh category, ‘Other’ (see Table 1, n = 5), was excluded; each of the five patients was admitted with a different working diagnosis. CI, confidence interval; CRP, C-reactive protein; ED, emergency department; HR, heart rate; OR, odds ratio; SBP, systolic blood pressure; UTI, urinary tract infection.

**Table 4:** Univariate analysis of post-discharge diagnosis by trajectory (not hospitalized vs. hospitalized). Three diagnoses reached significance individually: urosepsis (protective), bacteremia from an abdominal source, and endovascular infection (five patients with endocarditis and four with line sepsis).

Post-Discharge Diagnosis	Not Hospitalized (n = 114)	Hospitalized (n = 67)	OR	95% CI	*p* Value
Urosepsis	71 (62.3%)	24 (35.8%)	0.34	0.2–0.6	0.0007
Skin, soft-tissue, or bone infection	17 (15.0%)	17 (25.4%)	1.94	0.9–4.0	0.1
Abdominal infection	11 (9.6%)	14 (20.9%)	2.47	1.0–5.8	0.045
Pneumonia	9 (8.0%)	2 (3.0%)	0.36	0.1–1.7	0.2
Endovascular infection *	0 (0.0%)	9 (13.4%)	NA	NA	0.0001
Brucellosis	3 (2.6%)	1 (1.5%)	0.56	0.06–5.5	1.0
Primary bacteremia	3 (2.6%)	0 (0.0%)	0.00	NA	0.3

CI, confidence interval; NA, not applicable; OR, odds ratio. * Odds ratio not estimable due to zero events in the comparator group; Fisher’s exact test confirms the association is unlikely to reflect chance.

**Table 5:** Multivariable model of index ED visit predictors of return and hospitalization.

Variable	Adjusted OR	95% CI	*p* Value
Age (per 10 years)	0.97	0.8–1.1	0.67
Previous hospitalization, past 6 months	1.82	0.8–4.1	0.14
Creatinine ≥ 2 mg/dL	2.65	0.9–7.5	0.07
Non-UTI (1st ED discharge diagnosis)	2.53	1.3–5.0	0.007

CI, confidence interval; ED, emergency department; OR, odds ratio; UTI, urinary tract infection.

## Data Availability

The data presented in this study are available on reasonable request from the corresponding author. The data are not publicly available due to privacy restrictions.

## Abbreviations

The following abbreviations are used in this manuscript:

aOR	Adjusted odds ratio
AUC	Area under the receiver operating characteristic curve
CI	Confidence interval
CoNS	Coagulase-negative staphylococci
CRP	C-reactive protein
ED	Emergency department
ID	Infectious diseases
IQR	Interquartile range
MALDI-TOF	Matrix-assisted laser desorption/ionization time-of-flight
OR	Odds ratio
ROC	Receiver operating characteristic
SZMC	Shaare Zedek Medical Center
UTI	Urinary tract infection

## Appendix A

**Table A1:** Discharge diagnosis accuracy among patients who were discharged with occult bacteremia.

Working Diagnosis Outcome	N (%)	Not Hospitalized	Hospitalized	Hospitalization Rate
Correct diagnosis	107 (59.1%)	81	26	24.3%
No or incorrect working diagnosis	74 (40.9%)	33	41	55.4%
